# The Pap smear screening as an occasion for smoking cessation and physical activity counselling: baseline characteristics of women involved in the SPRINT randomized controlled trial

**DOI:** 10.1186/1471-2458-11-906

**Published:** 2011-12-07

**Authors:** Elisabetta Chellini, Giuseppe Gorini, Giulia Carreras, Livia Giordano, Emanuela Anghinoni, Anna Iossa, Cristina Bellati, Elisa Grechi, Alessandro Coppo, Fiorella Talassi, Maria Rosa Giovacchini

**Affiliations:** 1 Cancer Prevention and Research Institute, Florence, Italy; 2 CPO Piedmont, Turin, Italy; 3 Local Health Authority, Mantua, Italy; 4 Italian League against Cancer, Florence, Italy; 5 Local Health Authority, Florence, Italy; 6 Unit of Environmental and Occupational Epidemiology, Cancer Prevention and Research Institute (ISPO), Via delle Oblate 2, 50141 Florence, Italy

## Abstract

**Background:**

Gender-specific smoking cessation strategies have rarely been developed. Evidence of effectiveness of physical activity (PA) promotion and intervention in adjunct to smoking cessation programs is not strong. SPRINT study is a randomized controlled trial (RCT) designed to evaluate a counselling intervention on smoking cessation and PA delivered to women attending the Italian National Health System Cervical Cancer Screening Program. This paper presents study design and baseline characteristics of the study population.

**Methods/Design:**

Among women undergoing the Pap examination in three study centres (Florence, Turin, Mantua), participants were randomized to the smoking cessation counselling [S], the smoking cessation + PA counselling [S + PA], or the control [C] groups. The program under evaluation is a standard brief counselling on smoking cessation combined with a brief counselling on increasing PA, and was delivered in 2010. A questionnaire, administered before, after 6 months and 1 year from the intervention, was used to track behavioural changes in tobacco use and PA, and to record cessation rates in participants.

**Discussion:**

Out of the 5,657 women undergoing the Pap examination, 1,100 participants (55% of smokers) were randomized in 1 of the 3 study groups (363 in the S, 366 in the S + PA and 371 in the C groups). The three arms did not differ on any demographic, PA, or tobacco-use characteristics. Recruited smokers were older, less educated than non-participant women, more motivated to quit (33% vs.9% in the Preparation stage, *p *< 0.001), smoked more cigarettes per day (12 vs.9, *p *< 0.001), and were more likely to have already done 1 or more quit attempts (64% vs.50%, *p *< 0.001). The approach of SPRINT study appeared suitable to enrol less educated women who usually smoke more and have more difficulties to quit.

**Trial registration number:**

ISRCTN: ISRCTN52660565

## Background

Smoking is the leading cause of death and of many diseases for both men and women [1]. Since 1980, several surveys carried out by the Italian Institute of Statistics (ISTAT) showed a lower smoking decreasing trend in women (from 19.2% in 1986 to 17.0% in 2009) in comparison to men (from 41.6% in 1986 to 29.5% in 2009), and a younger age of initiation in girls, suggesting the need for gender-specific tobacco control strategies [2]. The Framework Convention on Tobacco Control also called for gender-specific prevention strategies [3]. However, these strategies, in particular on smoking cessation, have rarely been developed, except those for pregnant women [4], also in Italy [5].

The National Health System Cervical Cancer Screening Program (NHS-CCSP) in Italy is a beneficial setting to deliver smoking cessation counselling to women aged 25-64 years attending the Pap test examination. Smoking is an important co-factor for the development of cervical cancer, being human papilloma-virus (HPV) the principal causal factor [6]. Recent studies showed a synergistic effect between HPV-16 and smoking in the development of cervical carcinoma in situ [7]. HPV-positive smoking women are at higher risk of developing cervical intraepithelial neoplasia grade III (CIN3) [8,9]. Eighty percent of sexually active women contracts the infection up to 50 years of age [10]. Therefore, an intervention to reduce smoking prevalence in women should be a priority. In addition, women participating in screening programs are probably more sensitive to health promotion messages and more motivated to adopt risk-reducing health behaviours. For these reasons the screening settings might represent an occasion to promote healthy lifestyles, and this primary prevention activity might be easily integrated with the ongoing routine secondary prevention practice.

The linkage between smoking cessation and the fear to gain weight is well documented. Women's concern about their weight has been hypothesized to be a factor in preventing smoking cessation efforts, increasing smoking relapse rates among quitters, and encouraging smoking initiation. Women are more likely than men to believe that smoking helps control body weight and are more likely to report actually using cigarettes as a means to control their weight [11]. Because calorie reduction may enhance the reinforcing value of smoking, clinical practice guidelines recommended physical activity (PA), rather than diet [4,12]. Relatively small doses of exercise should be recommended as an aid to managing cigarette cravings and withdrawal symptoms [13]. Physical activity programs have been proposed as adjuncts to smoking cessation programs and to relapse prevention programs. However, a Cochrane review of 13 randomized controlled trials examining PA as a support for smoking cessation concluded that there was limited evidence that it helped [14]. Only one of the 13 trials found evidence for PA, in particular vigorous exercise, aiding smoking cessation at long term follow-up, with at least a delay in weight gain [15]. All the other trials were too small to exclude reliably an effect of intervention. There is also preliminary indication that even moderate-intensity exercise may enhance short-term smoking cessation outcomes for women [16]. A relapse prevention intervention showed that increased moderate to vigorous PA significantly predicted sustained 6-month abstinence [17]. Moreover, minimal interventions strategy aimed at promoting moderate/intensity PA with advice provided by health professionals, based on the trans-theoretical model of behavioural change [18], was effective in producing short-term increases in number of minutes walked among inactive individuals contemplating changes in their PA levels [19,20]. Therefore, adding a PA counselling to the standard smoking cessation counselling delivered may improve quit rates among women attending the Pap test examination.

The SPRINT Study was designed in order to verify the effectiveness of a standard counselling intervention on smoking cessation delivered by trained midwives in a gender-specific setting (the outpatient cervical cancer screening visits), and whether the adjunct of PA counselling to the standard smoking cessation counselling might increase quit rates among women undergoing the NHS-CCSP. This paper describes the characteristics of the female smokers enrolled in the study in comparison to those registered in non-participant female smokers.

## Methods/Design

### The SPRINT intervention

The Sprint intervention is a standard brief counselling on smoking cessation combined with a brief counselling on increasing PA. The smoking cessation counselling corresponds to the first two phases of the brief intervention for smoking cessation ("Ask" and "Advice") [12]. It is tailored according to the Di Clemente-Prochaska stage of change on smoking behaviour [18], and it takes about 2-3 min. The PA counselling is a brief intervention, designed and validated by psychologists involved in the study [19]. It is a stage-specific counselling to quit smoking, according to the Di Clemente-Prochaska stage assessment on PA [21]. The stages of change of each participant for smoking cessation and increasing PA were obtained from the study questionnaire. Counselling on smoking cessation and PA was delivered by trained midwives after the Pap smear. The involved midwives were specifically trained on motivational counselling on smoking cessation and increasing PA. A self-help booklet on smoking cessation and increasing PA was provided to all study participants.

### Study design

**Figure 1:**
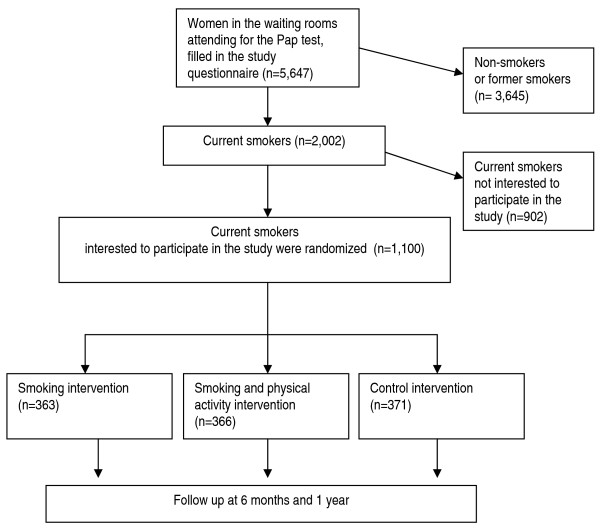
**Flow chart of the enrolment of women in the SPRINT study**.

IT is a three-arm randomized controlled trial. Smokers attending the NHS-CCSP consulting rooms who decided to participate in the study, were the units of analysis and were randomly assigned to one of the two intervention arms (smoking cessation counselling [S] arm, and the smoking cessation + PA counselling [S + PA] arm) or the control [C] arm (Figure 1).

### Units and subjects

The study population, consisting of women 25-64 years of age attending some NHS-CCSP consulting rooms, was expected to be enrolled in three Italian areas (4 consulting rooms in Florence, Tuscany, 3 in Turin, Piedmont, and 27 in Mantua, Lombardy). Women waiting to perform the test were asked to fill in the study questionnaire and, if participating in the study, the informed consent.

### Sample size

Assuming a six-month cessation rate in the S + PA arm of about 15%, and a cessation rate in the C group of about 8% (relative risk of 1.8), with a significant level α = 0.05, and a power of 0.80, a sample size of about 430 women per arm, and about 1,300 women overall, were estimated to be necessary to conduct the study. Cessation rates used in the power calculation derived from a pilot study previously conducted in one NHS-CCSP consulting room in Florence [22]. Knowing the number of women attending the NHS-CCSP annually in each consulting room of the 3 study centres, the smoking prevalence in 25-64 aged women of Northern-Central Italy (around 25% [2]), and the participation rate of about 30% observed in the above mentioned pilot study [22], we estimated the number of participants for the 3 centres.

### Selection and randomization of women

All smoking women, aged 25-64 years and attending the NHS-CCSP consulting rooms in the study areas, irrespective of their health status, were eligible if they consented to participate in the study. Participating women were randomly assigned to one of the 3 arms of the study by the midwives, using a predefined list of casual numbers.

### Outcome assessment

Participants had to fill in a questionnaire before to perform the test. Questions covered demographic characteristics, lifetime and current use of cigarettes, frequency of previous quit attempts, the Fagerstrom Tolerance Questionnaire (FTQ) [23], and intention to quit in order to assess the Di Clemente-Prochaska stage of change for smoking cessation [18]: from pre-contemplation phase to preparation phase. Non-participants had not to fill in the FTQ. In the questionnaire there were also questions regarding physical exercises. A 5-item scale was used to assess stage of change from sedentariness to regular and vigorous PA [21]: a total of 5 stages were distinguished in the questionnaire, following the Di Clemente-Prochaska stage of change (precontemplation, contemplation, preparation, action, or maintenance), and according to the intention to increase or maintain the frequency of PA at baseline PA levels (whether participants engaged in moderate-intensity PA for at least 30 min. on ≥ 5 days per week [24]).

A telephone follow-up to all participants was scheduled after six months and one year from the intervention, in order to know how many women successfully quitted after the intervention. The same questionnaire for tobacco use and PA levels was used in the follow-up interviews, in order to assess the Di Clemente-Prochaska stages of change for smoking cessation and increasing PA after the intervention. The analysis of these data are ongoing.

### Ethical aspects

The SPRINT Study was submitted and approved by the Ethics Committee of the Local Health Authority of Florence, Italy.

### Analysis

We conducted a descriptive analysis of the baseline characteristics of non-participant female smokers vs. recruited female smokers and across the study arms and centres. Differences in proportions were analyzed using the Chi-squared test. The non-parametric Mann-Whitney *U *test was used to test for difference in continuous variables, since data showed a non-normal distribution.

### Characteristics of the enrolled population

A total of 5,647 women who underwent the Pap smear test filled in the study questionnaire; 2,002 of them (35.4%) were current smokers, and 1,100 of them (54.9% of smokers) decided to participate to the study. Then, 363, 366, and 371 participants were randomly assigned to the S, S + PA, and C arms, respectively (Figure 1).

Recruited smokers were older, less educated, more likely to be married and employed (Table 1). Participation rate was significantly higher in Mantua (65%) than those recorded in Florence (53%) and Turin (50%). Moreover, recruited women smoked more cigarettes per day (12 vs. 9, *p *< 0.001), and were more likely to have already done ≥ 1 quit attempts than non-participants (64% vs. 50%; *p *< 0.001). Finally, looking at the Di Clemente-Prochaska stages of change for smoking cessation, recruited women were more likely to be in the preparation and contemplation stages, whereas non-participating women were more likely to be in the precontemplation stage (Table 1). Finally, 45% of recruited women engaged in moderate or intense PA for at least 30 min. on ≥ 5 days per week, 21% of participants were in the preparation stage for increasing PA, and 45% in the action or maintenance stages. Looking at the FTQ, 23% of participants recorded high or very high nicotine dependence.

**Table 1:** Comparison of baseline characteristics of non-participant women vs. recruited women, by SPRINT trial arm (C: Control arm, S: Smoking intervention, S + PA: Smoking and physical activity intervention)

	Non participant women (*n *= 902)	Recruited women (*n *= 1,100)	*p*-value	C (*n *= 371)	S (*n *= 363)	S + PA (*n *= 366)
Age (years) - mean (sd)	42 (11.8)	43 (10.8)	0.005	43 (10.3)	43 (11.2)	44 (11.0)

Education: graduated - *n *(%)	218 (24.2)	204 (18.5)	0.002	68 (18.3)	70 (19.3)	66 (18.0)

Marital status - *n*(%)			0.006			

Never married	296 (32.8)	280 (25.4)		90 (24.3)	98 (27.0)	92 (25.1)

Married	471 (52.2)	616 (56.0)		219 (59.0)	194 (53.7)	202 (55.2)

Divorced/separated	105 (11.6)	163 (14.8)		49 (13.2)	58 (16.0)	56 (15.3)

Widowed	22 (2.4)	30 (2.7)		9 (2.4)	8 (2.2)	13 (3.5)

Women with children - *n *(%)	282 (31.3)	387 (35.2)	0.169	139 (37.5)	122 (33.6)	126 (34.4)

Employed - *n *(%)	600 (66.5)	773 (70.3)	0.048	258 (69.5)	258 (71.1)	257 (70.2)

Study centre - *n *(%)			< 0.001			

Florence, Tuscany	194 (47.4)	215 (52.6)		72 (19.4)	73 (20.1)	70 (19.1)

Turin, Piedmont	495 (50.3)	489 (49.7)		165 (44.5)	161 (44.3)	163 (44.5)

Mantua, Lombardy	213 (35.0)	396 (65.0)		134 (36.1)	129 (35.5)	133 (36.3)

Physical activity characteristics						

30 min. of moderate/intense PA ≥ 5 days a week - *n *(%)	440 (48.8)	499 (45.4)	0.146	177 (47.7)	155 (42.7)	167 (45.6)

Stage - *n*(%)			0.318			

Precontemplation/Contemplation	212 (23.5)	286(26.0)		99 (26.7)	92(25.3)	95(26.0)

Preparation	171(19.0)	227(20.6)		69(18.6)	82(22.6)	76(20.8)

Action/Maintenance	440(48.8)	499(45.4)		177(47.7)	155(42.7)	167(45.6)

Missing	79 (8.8)	88 (8.0)		26(7.0)	34(9.4)	28(7.6)

Smoking habits characteristics						

Cigarettes per day - mean (sd)	9(6.3)	12 (7.1)	< 0.001	13 (7.7)	12 (6.8)	12 (7.0)

≥ 20 cigarettes per day - *n*(%)	87 (9.6)	230 (20.9)	< 0.001	87 (23.4)	71 (19.6)	72 (19.7)

Age of smoking initiation -mean (sd)	19 (5.0)	18 (5.0)	0.142	18 (6.5)	18 (4.7)	19 (5.3)

≥ 1 quit attempts - *n*(%)	454 (50.3)	708 (64.4)	< 0.001	250 (67.4)	242 (66.7)	216 (59.0)

Stage (%)			< 0.001			

Precontemplation	556 (61.6)	338 (30.7)		113(30.5)	102(28.1)	123(33.6)

Contemplation	199 (22.1)	353 (32.1)		128(34.5)	110(30.3)	115(31.4)

Preparation	84 (9.3)	359 (32.6)		117(31.5)	130(35.8)	112(30.6)

Missing	63(7.0)	50(4.5)		13(3.5)	21(5.8)	16(4.4)

Fagerström Test for Nicotine Dependence						

High-very high dependence - *n*(%)	--§	254 (23.1)		99 (26.7)	87 (24.0)	68 (18.6)

Smoking the first cigarette within 5 min. after awakening- *n *(%)	--§	120 (10.9)		45 (12.1)	39 (10.7)	36 (9.8)

§ Non participant women did not fill in the Fagerstrom Questionnaire

There were no significant differences among the 3 arms of the study, looking at socio-demographic, PA, and tobacco-use variables (Table 1).

## Discussion

The SPRINT Study is an Italian multicenter trial aiming to evaluate the effectiveness of a NHS-CCSP-based smoking cessation intervention with an additional counselling on PA. It mobilizes resources from 3 regional centres in Italy, and involved 1,100 women.

The cervical cancer screening programme represents an opportunity to contact a large number of smoking women: in the last years, in Italy, more than 1,500,000 women attend the initial examination (the Pap smear), [25] and 19.6% out of them are estimated to be smokers [26].

The three study centres (Florence, Turin, and Mantua) recruited the expected study samples, and recorded a participation rate from 50% in Turin to 65% in Mantua, higher than the participation rate of 31.5% recorded in the pilot study previously conducted [22]. This high participation rate might be due to the high sensitivity to smoking cessation of recruited women and, at the same time, to the direct involvement in the study of trained midwives of the outpatient cervical cancer screening visits, meanwhile in the first pilot study the recruitment was carried out by an health visitor in the waiting room for the Pap test examination.

Other strengths of the study are the large sample size and the measurement of variations from baseline stage of self-reported behavioural change for smoking cessation and increasing PA after the intervention.

Recruited women recorded peculiar tobacco-use characteristics in comparison to non-participant women: they were more motivated to quit (about one third was in the preparation stage), smoked more cigarettes per day, and had already attempted to quit. Eighty-one percent of smokers in the preparation stage for smoking cessation, 64% in the contemplation stage, and only 38% in the pre-contemplation stage participated voluntarily to the study. Thus, this distribution by motivation level among participants could determine an unexpectedly high cessation rate in the control group. Anyhow, this bias is unavoidable in intervention studies where the intervention cannot be imposed and the subjects under study must be necessarily volunteers.

Another limit of the study is that all the outcome measures on smoking cessation and PA are self-reported, even though self-reports are considered a low-cost approach to obtaining sufficiently accurate information on tobacco use and PA [27,28].

Regarding the motivational phase, it must be outlined that for PA the percentage of sedentary women in pre-contemplation (those who does not want even to think to change their habit) is similar in recruited and in non participant women (almost 25%); on the contrary pre-contemplating smokers among non participants are twice the participants (61.6% vs 30.7%). These data reflect the high attention of women on their body weight and at the same time the resistance to quit. Smoking appeared to attenuate weight gain over time and this is also a reason of female smokers' resistance to quit smoking, even if the average weight of women who are current smokers is modestly lower than that of women never smoked or who are former smokers [4-29].

In conclusion, smoking is actually the predominant health problem in developed countries, accounting for about 19,000 attributable deaths in Italian women in 2010 [30]. Promoting smoking cessation in women is one of the most important strategies to reduce female smoking attributable mortality in next decades. In order to meet this challenge, SPRINT trial uses a gender-specific setting to deliver smoking cessation counselling to women, and evaluates the effectiveness of a standard smoking cessation counselling intervention tailored to women with an adjunct of PA counselling. Moreover, through the study approach it was possible to enrol less educated women who usually smoke more and have more difficulties to quit.

## Competing interests

The authors declare that they have no competing interests.

## Authors' contributions

EC conceived of the study, participated in the design of the study, coordinated the multicentric study group and draft the manuscript. GG participated in the design and analysis of the study, and contributed to the manuscript draft. GC analysed the data and contributed to the manuscript draft. LG participated in the design of the study, was responsible of the study in Piedmont, and contributed to the manuscript draft. EA participated in the design of the study, was responsible of the study in Lombardy, and contributed to the manuscript draft. AI participated in the design of the study, was responsible of the study in Tuscany, and contributed to the manuscript draft. CB participated in the design of the study and trained the personnel involved in recruitment. EG participated in the design of the study, trained the personnel involved in recruitment, and revised the manuscript. AC participated in the design of the study, carried out the study in Piedmont and revised the manuscript. FT participated in the design of the study, carried out the study in Lombardy and revised the manuscript. MRG participated in the design of the study, carried out the study in Tuscany and revised the manuscript. All authors read and approved the final manuscript.

## Author's information

The SPRINT Working Group includes the previous mentioned authors and all the midwives who enrolled the population under study: E. Amadori (Local Health Authority of Mantua, Italy); E. Anghinoni (Local Health Authority of Mantua, Italy); A.M. Badiali (ISPO, Florence, Italy); B. Baldini (Local Health Authority of Florence, Italy); B. Baluga (Local Health Authority of Mantua, Italy); A.M. Barbi (Local Health Authority of Mantua, Italy); M.C. Barbieri (Local Health Authority of Florence, Italy); C. Bellati (CPO, Turin, Piedmont, Italy); B. Benatti (Local Health Authority of Mantua, Italy); E. Berini (Local Health Authority of Mantua, Italy); L. Boldrini (Local Health Authority of Mantua, Italy); P. Breviglieri (Local Health Authority of Mantua, Italy); C. Bronchi (Local Health Authority of Florence, Italy); V. Cacciarini (ISPO, Florence, Italy); L. Calabrese (Local Health Authority of Florence, Italy); L. Campitelli (Local Health Authority of Mantua, Italy); L. Caraffa (Local Health Authority of Mantua, Italy); N. Casoni (Local Health Authority of Mantua, Italy); G. Catelani (Local Health Authority of Florence, Italy); D. Casi (Local Health Authority of Florence, Italy); P. Cavini (Local Health Authority of Florence, Italy); E. Chellini (ISPO, Florence, Italy); S. Clara (Local Health Authority of Turin, Italy); A. Cerchi (Local Health Authority of Turin, Italy); N. Colledan (Local Health Authority of Turin, Italy); A. Coppo (CPO, Turin, Piedmont, Italy); L. Cozzi (Local Health Authority of Florence, Italy); C. Danielis (Local Health Authority of Mantua, Italy); C. Di Pierro (ISPO, Florence, Italy); F. Di Stefano (CPO, Turin, Piedmont, Italy); C. Ferrari (Local Health Authority of Mantua, Italy); M. Ferri (Local heath Authority of Mantua, Italy); T. Ferri (Local Health Authority of Mantua, Italy); L. Fiaccadori (Local Health Authority of Mantua, Italy); F. Florio (Local Health Authority of Mantua, Italy); C. Forlucci (Local Health Authority of Florence, Italy); E. Galanti (Local Health Authority of Florence, Italy; A.M. Gallina (Local Health Authority of Mantua, Italy); M. Gialdini (Local Health Authority of Mantua, Italy); L. Giordano (CPO, Turin, Piedmont, Italy); M.R. Giovacchini (Local Health Authority of Florence, Italy); G. Gorini (ISPO, Florence, Italy); E. Grechi (LILT, Florence, Italy); L. Grossi (Local Health Authority of Mantua, Italy); S. Guzzo (Local Health Authority of Mantua, Italy); G. Innocenti (Local Health Authority of Florence, Italy); A. Iossa (ISPO, Florence, Italy); S. Labardi (Local Health Authority of Florence, Italy); C. Lepri (Local Health Authority of Florence, Italy); D. Montovanelli (Local Health Authority of Mantua, Italy); G. Mantovani (Local Health Authority of Mantua, Italy); A. Marchi (Local Health Authority of Mantua, Italy); S. Mazzoni (Local Health Authority of Florence, Italy); M. Molinari (Local Health Authority of Mantua, Italy); M. Morandini (Local Health Authority of Florence, Italy); R. Nidiaci (ISPO, Florence, Italy); P. Noli (Local Health Authority of Mantua, Italy); A.M. Notarangelo (Local Health Authority of Turin, Italy); S. Oliveri Del Castillo (Local Health Authority of Mantua, Italy); R. Pasini (Local Health Authority of Mantua, Italy); F. Perfetti (Local Health Authority of Mantua, Italy); C. Prati (Local Health Authority of Mantua, Italy); G. Pria (Local Health Authority of Mantua, Italy); S. Raffanini (Local Health Authority of Mantua, Italy); N. Rigoni (Local Health Authority of Mantua, Italy); E. Russo (Local Health Authority of Mantua, Italy); R. Sgarbi (Local Health Authority of Mantua, Italy); M.E. Siliprandi (Local Health Authority of Mantua, Italy); D. Simoncelli (Local Health Authority of Mantua, Italy); F. Talassi (Local Health Authority of Mantua, Italy); C. Ticci (Local Health Authority of Florence, Italy); S. Toffalini (Local Health Authority of Mantua, Italy); C. Tovagliari (Local Health Authority of Mantua, Italy); M. Vaccari (Local Health Authority of Mantua, Italy); C. Vair (Local Health Authority of Turin, Italy), M. Zambello (Local Health Authority of Mantua, Italy).

## Pre-publication history

The pre-publication history for this paper can be accessed here:

http://www.biomedcentral.com/1471-2458/11/906/prepub
